# Supplementary material for: Genetic Relationships of Puccinia striiformis f. sp. tritici in Southwestern and Northwestern China
Source: Microbiol Spectr. 2022 Jul 27;10(4):e01530-22. doi: 10.1128/spectrum.01530-22 (PMC9430570; doi:10.1128/spectrum.01530-22)
Supplement: Supplemental file 1 — Supplemental material. Download spectrum.01530-22-s0001.pdf, PDF file, 6.6 MB [file spectrum.01530-22-s0001.pdf]

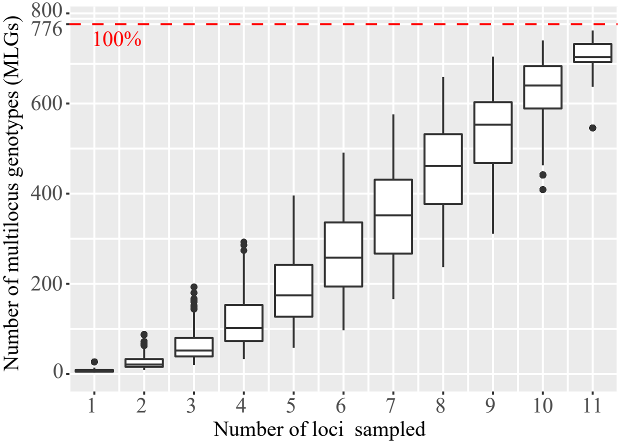

FIG S1 Genotype accumulation curve reflecting whether loci are sufficient for population analysis. The Genotype Curve reached 100%, indicating that the number of loci selected was sufficient for population analysis. In this study, 11 pairs of markers were enough to identify all of the *Pst* isolates.

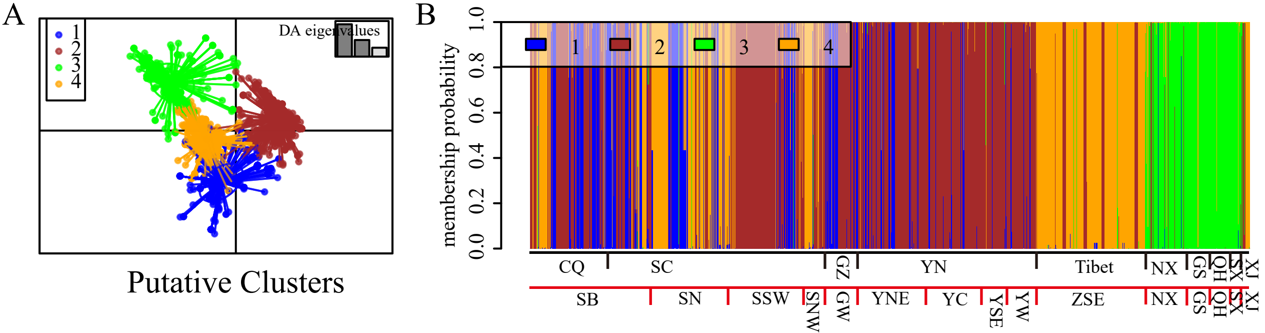

FIG S2 Assignment of isolates determined using DAPC when the putative cluster was 4, showing partial overlap.

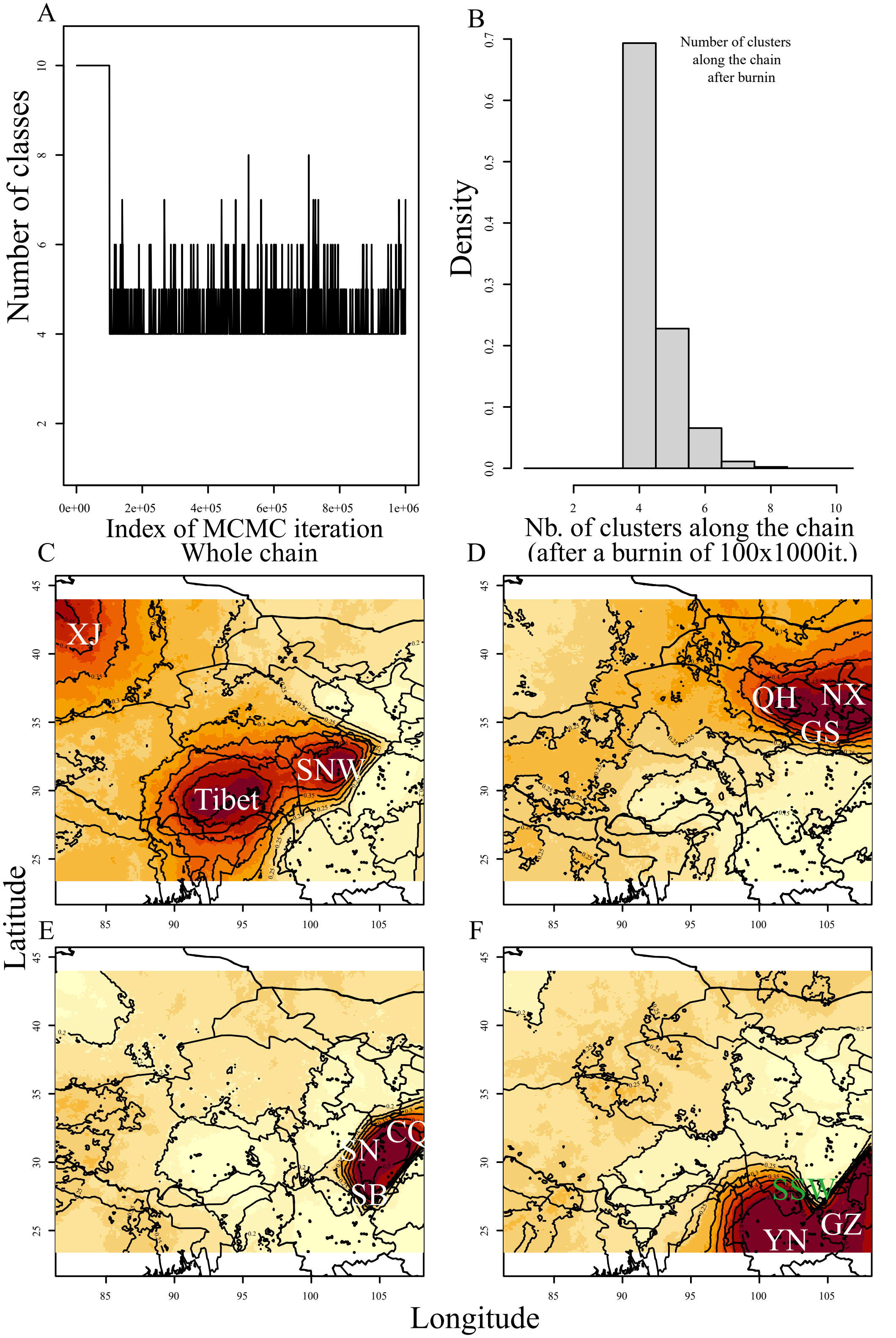

FIG S3 Maps of the posterior probabilities of population membership inferred by GENELAND. (A-B) The 300 *Pst* isolates inferred four clusters. (C-F) The genetic connectivity in the four clusters. Contour lines indicate the spatial position of the genetic discontinuities. The same shading indicates high probability similarities of population membership.
